# EFFECTS OF HIGH-INTENSITY AQUATIC EXERCISE ON INFLAMMATION AND COGNITION IN OLDER ADULTS WITH MCI

**DOI:** 10.1093/geroni/igad104.3293

**Published:** 2023-12-21

**Authors:** Peter Louras, Adriana Savettiere, Windy McNerney, Kaci Fairchild

**Affiliations:** VA Palo Alto Healthcare System, Palo Alto, California, United States; Palo Alto University, Palo Alto, California, United States; VA Palo Alto Healthcare System, Palo Alto, California, United States; VA Palo Alto Healthcare System, Palo Alto, California, United States

## Abstract

Exercise shows immune benefits in older adults and can reduce the risk of late-life cognitive impairment and dementia. This study sought to explore the effects of high-intensity aquatic-based exercise on older adults with mild cognitive impairment (MCI), specifically by examining changes in inflammatory plasma protein concentrations and cognitive performance. A secondary data analysis was conducted on 20 healthy volunteers (mean age 68.2 ±7.75 years) who participated in 6 months of supervised aquatic exercise training. Participants completed a battery of neuropsychological assessments and whole blood draws at pre- and post-intervention. Plasma protein concentration levels were attained using the Olink Target96 Inflammation panel [Uppsala, Sweden]. Mixed effects models assessed the effects of protein concentration change scores on selected cognitive outcomes, while controlling for age. Results indicated that following the exercise intervention, significant changes in two inflammatory-related proteins (Extracellular newly identified receptor for advanced glycation end-products binding protein [EN-RAGE]; Interleukin-20 receptor subunit alpha [IL-20RA]) were associated with improved verbal memory and processing speed endpoints. Specifically, decreased EN-RAGE concentrations resulted in significantly improved delayed verbal memory scores (parameter estimate= -0.893, p= .023), while increased IL-20RA concentrations led to better task completion (parameter estimate= 2.145, p= .030). These findings demonstrate the cognitive benefits of aquatic-based exercise for older adults and those with MCI, and suggest these effects are mediated by alterations in pro- and anti-inflammatory protein markers such as EN-RAGE and IL-20RA. Larger studies are warranted to replicate these findings, and to further explore the role of activity-induced inflammatory changes on late-life brain health.

